# Single-stage transplantation combined with epidermal stem cells promotes the survival of tissue-engineered skin by inducing early angiogenesis

**DOI:** 10.1186/s13287-023-03281-z

**Published:** 2023-03-23

**Authors:** Zhiyong Wang, Hailin Xu, Hao Yang, Yi Zhang, Xiaoyan Wang, Peng Wang, Zhongye Xu, Dongming Lv, Yanchao Rong, Yunxian Dong, Bing Tang, Zhicheng Hu, Wuguo Deng, Jiayuan Zhu

**Affiliations:** 1 Department of Burn and Wound Repair Surgery, First Affiliated Hospital of Sun Yat-Sen University, Guangzhou, China; 2 Collaborative Innovation Center of Cancer Medicine, State Key Laboratory of Oncology in South China, Sun Yat-Sen University Cancer Center, Guangzhou, China; 3 Department of Burn and Plastic Surgery, Affiliated Hospital of Nantong University, Nantong, China; 4 Department of Plastic Surgery, Guangdong Second Provincial General Hospital, Southern Medical University, Guangzhou, China

**Keywords:** Epidermal stem cells, Acellular dermal matrix, Tissue-engineered skin, Vascularization, Angiogenesis

## Abstract

**Background:**

The composite transplantation of a split-thickness skin graft (STSG) combined with an acellular dermal matrix (ADM) is a promising repair method for full-thickness skin defects. Due to delayed vascularization of the ADM, no currently available engineered skin tissue is able to permanently cover full-thickness skin defects via a single-stage procedure. Epidermal stem cells (EpSCs) have been found to promote angiogenesis in the wound bed. Whether EpSCs can induce early angiogenesis of dermal substitutes and promote the survival of single-stage tissue-engineered skin transplantation needs to be further studied.

**Methods:**

In vitro, rat vascular endothelial cells (RVECs) were treated with the supernatant of EpSCs cultured in ADM and stimulated for 48 h. RVECs were analysed by RNA sequencing and tube formation assays. For the in vivo experiment, 75 rats were randomly divided into five groups: ADM, ADM + EpSCs (AE), STSG, ADM + STSG (AS), and ADM + STSG + EpSCs (ASE) groups. The quality of wound healing was estimated by general observation and H&E and Masson staining. The blood perfusion volume was evaluated using the LDPI system, and the expression of vascular markers was determined by immunohistochemistry (IHC).

**Results:**

The active substances secreted by EpSCs cultured in ADM promoted angiogenesis, as shown by tube formation experiments and RNA-seq. EpSCs promoted epithelialization of the ADM and vascularization of the ADM implant. The ASE group showed significantly increased skin graft survival, reduced skin contraction, and an improved cosmetic appearance compared with the AS group and the STSG control group.

**Conclusions:**

In summary, our findings suggest that EpSCs promote the formation of new blood vessels in dermal substitutes and support one-step transplantation of tissue-engineered skin, and thereby provide new ideas for clinical application.

**Supplementary Information:**

The online version contains supplementary material available at 10.1186/s13287-023-03281-z.

## Background

Full-thickness skin defects caused by burns and wounds can lead to pathogen invasion, body temperature disorders, and dehydration, which can be life-threatening in severe cases. Achieving rapid healing of full-thickness wounds is a critical need and remains a great clinical challenge [1]. There is no doubt that autologous full-thickness skin grafts (FTSGs) are the most appropriate treatment for skin injuries [2]. Although FTSGs can replace the full thickness of the skin, their application remains limited due to insufficient donor skin sources and wound bed contamination. Autologous split-thickness skin grafts (STSGs) are currently the most commonly used method in clinical practice due to the advantages of repeated collection from donor sites. However, STSGs lack dermal components; thus, these grafts are more fragile and undergo significantly more shrinkage compared with FTSGs, and these features affect cosmesis during the healing stage.

Acellular dermal matrix (ADM) is a cell-free three-dimensional reticular biological scaffold that can not only induce cell recolonization to promote regeneration in vivo, but also implant the recipient site to provide sufficient dermal tissue to the wound surface and greatly reduce the degree of scar formation and wound contracture. Therefore, ADM grafts can be combined with autologous STSGs to overcome the drawbacks of STSGs alone [3]. Currently, at least two approaches are needed; the dermal matrix is first implanted into the wound bed; the STSGs are then implanted until the ADM is fully revascularized by attracting cells and growth factors; and the interval between cycles is generally 2 to 4 weeks [5], which leads to a lengthy repair time and increased financial costs. Nevertheless, no currently ADM and STSGs can permanently cover full-thickness skin wounds via a single-stage transplantation. These composite transplants may undergo degeneration and necrosis due to early hypervascularity followed by a long period of ischaemia and hypoxia in single-stage procedures [4]. Acquiring permanent regenerative repair in full-thickness skin defects remains a clinical challenge.

Delayed vascularization is a significant barrier to developing and applying dermal substitutes to the skin. Various methods have been established to promote rapid vascularization of dermal substitutes, and these include thinning, structural optimization [5–7], and the introduction of vasoactive factors [8, 9]. Undoubtedly, a thicker ADM is associated with the need for a longer vascularization time. A thinner dermal scaffold achieves survival of superficial STSGs, whereas degradation occurs before collagen deposition and neovascularization [10]. Scaffold-induced vascularization is limited by alterations in the ADM structure (i.e. pore size, porosity, and connectivity). Introduced vasoactive factors also undergo degradation exceptionally rapidly within a short period, which leads to the need for multiple doses. From angiogenesis to maturation, the vascularization process in the dermal substitute is tightly regulated by various cellular and bioactive factors [11]. Therefore, enhancing the vascularization of tissue-engineered skin via the delivery of cells and the release of bioactive factors is an emerging strategy for the promotion of wound healing [12, 13].

Stem cells are considered ideal seed cells for promoting wound healing [14–16]. Stem cells have multilineage differentiation capacity and secrete various bioactive factors according to the microenvironment [17, 18]. Many studies have used stem cells for the treatment of skin wounds. Stem cells can be applied directly to wounds or combined with dermal substitutes. At present, there are many reports in the literature that adipose-derived mesenchymal stem cells (ASCs) and dermal-derived mesenchymal stem cells (DSCs) promote vascularization in wounds [19, 20]. These cells can promote the vascularization of wound tissue and dermal substitutes. Epidermal stem cells (EpSCs) distributed in the basement layer of the epidermis in tissue-engineered skin are relatively rare [4]. The more EpSCs that remain on the wound skin, the faster the wounds heal, and less scarring occurs. Theoretically, any skin wound can rely on EpSCs for physiological healing. However, residual skin stem cells cannot usually differentiate for more extensive full-thickness skin wounds to complete the skin’s anatomical structure and functional repair due to the loss of skin stem cells. The healing process may be uncontrolled, which can eventually lead to the formation of scar tissue without hair and sweat glands [4, 21]. Furthermore, new studies have utilized native ECM culture systems to model the stem cell niche, which is essential for the maintenance of EpSC homeostasis [22]. Thus, many recent studies have utilized natural biopolymers as matrices for the delivery of stem cells in vivo.

Our previous study showed that EpSCs could proliferate and differentiate in the wound bed and observed that EpSCs form new blood vessels in the wound bed [23]. We hypothesized that the vascularization process inside the dermal substitutes implanted into the wound was similar to that in the site of damage. This study investigated whether EpSCs could rapidly promote vascularization of the ADM and promote wound healing. We then innovatively constructed a novel tissue-engineered skin using a combination of ADM, an STSG, and EpSCs (ASE) for a single-stage procedure to cover a full-thickness skin wound in vivo. Improvements in the healing rate and quality of the wound skin and a shortened treatment cycle were observed. Moreover, the specific mechanism through which this construct promoted healing and vascularization was explored to provide a preliminary scientific basis for its clinical application.

## Materials and methods

### Material preparation

The ADM sheets of HEAL-FULL® was provided by Yantai Zhenghai Biotechnology Co. (20,143,462,108, Shandong, China). Briefly, the epidermal layer and subcutaneous fat were first removed from skin obtained from healthy bovines. The 0.4-mm split-thickness skin graft was manufactured. Sodium hydroxide and trypsin were used to remove cell components, which were washed with in purified water until the water was completely clear. The ADM sheet was then lyophilized and sterilized with Co (15 kGy) before packaging for future use. The ADM sheet is un-cross-linked. The ADM sheets were cut into discs with a diameter of 6 mm using a punch (Deli, China) in vitro and discs with a diameter of 25 mm in vivo.

### Gross view and scanning electron microscopy (SEM)

To observe the morphology of the ADM, we recorded the gross characteristics of the material with a single reflection camera (Nikon, Japan). The surface morphology of the dried samples was scanned by scanning electron microscopy (Zeiss Gemini 300, Germany).

### Fourier transform infrared (FTIR) spectroscopy

The ADM was characterized by FTIR (TENSOR27 FTIR Spectrometer). In a dry atmosphere at room temperature, the transmission mode was intervals of 4 cm^−1^ in the wavelength range of 600–4000 cm ^−1^.

### Tensile strength

The tensile strength of the materials (*n* = 3) was measured using a mechanical analyser (model 1007–1601, Ningbo Weiheng, China). The ADM had a length of 100 mm, a width of 10 mm, and a height of 0.4 mm. The sample was stretched at a speed of 25 mm/min until it broke. The maximum load value at which the specimen broke was recorded.

### Isolation and culture of rat EpSCs

Three two-day-old rats were killed by cervical dislocation. The skin of the rats was collected, rinsed in 1% phosphate-buffered saline (PBS; 10,010,023; Gibco), and then placed on ice. The skin with the subcutaneous fascia removed was cut to a size of 1 × 1 cm^2^ and placed in a cell sorter (Guangzhou Myseed Medical Technology Co., Ltd.), and 2 mL of 10 × TrypLE (A1217702; Gibco) was added for digestion at 37 °C for 20 min such that the epidermis could be scraped off using a sterile scalpel. Moreover, several T25 culture flasks were evenly coated with 1 mL (0.5 mg/ml) of fibronectin (FN; 5 μg/cm^2^; Shanghai Fibronectin Biotechnology, Shanghai, China) solution before planting of the basal cell suspension and then incubated at 37 °C for 20 min. The epidermis was then collected and digested in 0.25% trypsin–EDTA solution for 5 min. The digested cell suspension was subsequently filtered into a 50-mL centrifuge tube using a 100 μm-mesh filter (BS-100-CS, Biosharp) at 1000 r/min for 10 min. The basal cell suspension was resuspended in keratinocyte serum-free medium (K-SFM; 17,005,042; Gibco), transferred into precoated culture flasks, and incubated at 37 °C for 20 min to replace the medium. EpSCs were cultured at 37 °C in a 5% CO_2_ atmosphere, and adherent cells were then cultured and expanded with K-SFM every 2-days. Cells at passage three were used for all experiments.

### Identification of rat EpSCs

Approximately 5,000 EpSCs per sample were seeded in cover-glass-bottomed dishes (801,002, NEST) and subjected to three 5-min washes with PBS after cell adhesion. After 24 h, the cell samples were fixed with 4% paraformaldehyde for 20 min at room temperature. The samples were washed three times with PBS, and then incubated with 0.5% Triton X-100 (P0096-500–50 mL; Beijing Biotechnology Company, China) for 20 min at room temperature. The samples were washed three times with PBS, 5% goat serum (C0265; Beijing Biotechnology Company, China) was then added to glass-bottomed dishes for blocking, and the samples were then incubated at room temperature for 40 min. Subsequently, the cells were incubated with p63 (1:200; ab735; Abcam), ITGα6 (1:200; ab235905; Abcam), and CK15 (1:200; ab80522; Abcam) at 4 °C overnight and then with the corresponding fluorescent secondary antibodies, namely Alexa Fluor 488-labelled goat anti-rabbit IgG (1:200; ab150077; Abcam), Alexa Fluor 594-labelled goat anti-mouse IgG (1:200; ab150116; Abcam), and goat anti-rat IgG HampLDyLight® 594 (1:200; ab96889; Abcam) at room temperature for 60 min. DAPI (D9542; Sigma-Aldrich) was used for nuclear counterstaining for 10 min in the dark. The samples were then subjected to three 5-min washes with PBS, fixed by dropwise addition of an anti-fluorescence quencher, and observed under a fluorescence microscope.

### Flow cytometry

EpSCs at passage 3 from three samples were harvested by centrifugation and then resuspended in 0.1 mL of PBS. Cell aliquots (1 × 10^6^ cells/mL) were stained with primary antibodies (K10, Novus, NBP2-61,736, 1:50; ITGα6, Abcam, ab77906, 1:50) on ice for 30 min. The cells were then rinsed with incubation buffer, centrifuged, and then incubated for 30 min on the ice with fluorescently labelled secondary antibodies diluted in incubation buffer as recommended by the manufacturer. The cells were subsequently rinsed with incubation buffer, centrifuged, resuspended in 0.5 mL of PBS, and then subjected to flow cytometry analysis.

### Lentivirus transfection

To trace the epidermal stem cells in vitro and in vivo, third-generation EpSCs at 50% confluency were labelled with enhanced green fluorescence protein (EGFP) using lentiviruses (MOI: 20) or an empty control (GeneChem, Shanghai, China) following the manufacturer’s instructions. Forty-eight hours after transfection, stable clones were selected using puromycin at a concentration of 2 µg/ml for 1 week. The EGFP expression level was determined by immunofluorescence.

### Cell viability and adhesion

In this study, 5 × 10^4^ EpSCs expressing EGFP were seeded on 6-mm-diameter round ADM fragments in 96-well plates and cultured for 24 h. The cells were gently rinsed with PBS to remove dead and nonadherent cells and counted as N for use in the following formula: cell adhesion rate on the scaffold = (total cell number-N)/total cell number × 100%. Moreover, the expression of EGFP in the ADM was visualized by fluorescence microscopy. To determine the viability of EpSCs in the ADM scaffolds, the cell viability and activity were assessed using the 3-(4,5-dimethyl methyl thiazole-2-yl)-2–5-diphenyl bromo tetrazolium method (MTT; Promega) according to the manufacturer’s instructions. After 1, 4, and 7 days of incubation, the absorbance was read using a plate reader (Tecan Infinite M200) at a wavelength of 490 nm.

### RNA sequencing analysis

Rat vascular endothelial cells (RVECs) (Jiangsu Sitai Experimental Equipment Co., LTD.) were stimulated with the supernatant (1 mL) of EpSCs cultured under different conditions (ADM sheets or control) for 48 h. Total RNA was extracted from RVECs using an RNA purification kit (R4013-03, Magen) following the manufacturer’s instructions. The transcripts per million reads were used for the calculation of gene expression, and genes with |log2 (fold change)|≥ 1 and *p* value < 0.05 were considered statistically significant. Kyoto Encyclopedia of Genes and Genomes (KEGG; http://www.genome.jp/kegg/) analyses were performed to elucidate the functions and enriched pathways of statistically significant genes.

### Tube formation assays

Matrigel matrix (356,234, Corning) was plated in 48-well culture plates (150 μL/well) and incubated at 37 °C for 20 min. RVECs were then inoculated at a density of 3 × 10^4^ cells/well with 1 mL of conditioned culture medium (Fig. 3A). Matrigel cultures were imaged at 8 h with a Nikon microscope. Images from each group were analysed using ImageJ software (Angiogenesis package). The number and length of branches were used for the quantitative analysis of angiogenesis.

### Surgical procedures in vivo

All experiments were conducted out in accordance with the Institutional Animal Care and Use Committee of the First Affiliated Hospital of Sun Yat-sen University (Ethics number 2020–448). Seventy-five adult male Sprague‒Dawley rats (200–220 g) were purchased from Zhuhai Bestest Biotechnology Co., Ltd. (Zhuhai, China). After being adapted to laboratory conditions for 1 week, the rats were anaesthetized by the inhalation of 1.5–3% isoflurane (INH). After the rats were shaved, scrubbed, disinfected, and covered with sterile draping, a 25-mm-diameter round full-thickness skin defect was made on the dorsal side of each rat. The defects were randomly divided into five groups and repaired with ADM, ADM + EpSCs (AE), STSG, ADM + STSG (AS), or ADM + STSG + EpSCs (ASE). The STSGs were obtained from the abdominal skin of autologous rats. The STSGs were obtained from the abdominal skin of autologous rats and yielding a final thickness of approximately 0.3 mm (Additional file 1: Fig. S1A, B). One millilitre of EpSCs expressing EGFP at a cell density of 1 × 10^6^/mL was evenly sprayed on the ADM bed in the AE and ASE groups (Additional file 1: Fig. S1F, G). The implants were fixed without tension by interrupted, nonresorbable packing via pressurized fixation with intermittent sutures of 5–0 nylon silk thread (Additional file 1: Fig. S1H–J). After recovery, each rat was returned to its cage and given free access to food and drinking water. Sutures in each group were uniformly removed at 1 week.

### Evaluation of skin wounds and blood perfusion

The rats and wounds were observed, and photographs of the dorsal skin were taken at the same location using a digital camera at each postoperative time point. Rats with poor damage due to fighting or wound infection were excluded. The wound photographs were blindly evaluated by two surgeons after comparing the patch colour changes, eschar, and epithelialization areas. At each sampling time point, ImageJ software (National Institutes of Health, Bethesda, MD, USA) was used to measure the total surface area and unhealed wound area, and the wound healing and contraction were calculated according to the following formulas: healing wound area rate (%) = [(the area at n weeks-unhealed area at n weeks)/(the area at n weeks)] × 100% and wound contraction rate (%) = [(the area at 0 weeks- the area at n weeks)/the area at 0 weeks] × 100%. Rats in each group were killed at the indicated time, the wound tissue was harvested, and the centre was divided into two portions: One-half was used for histology and immunohistochemistry analysis, and the other half was snap-frozen in liquid nitrogen for further analysis.

Simultaneously, the skin’s blood perfusion volume was evaluated using the LDPI system (PeriScan PIM3, Perimed, Stockholm, Sweden). The probe scanned the dorsal skin surface for at least 30 s, and the results were recorded as the blood relative perfusion unit (RPU) value. Contrast images were processed to display a colour-coded real-time flux image. To accurately quantify the range of the blood supply region, the latter was determined as an RPU value < 300.

### Immunohistochemical analysis

Three rats in each group were killed through the inhalation of excessive amounts of isoflurane at predetermined time points (3, 7, 14, 21, and 42 days). The grafts and surrounding tissues were harvested and divided into two parts along the centreline. All samples were fixed in 10% formalin at room temperature. Paraffin-embedded formalin-fixed tissue sections (5 μm) were dewaxed, dehydrated, and rehydrated with xylene and gradient ethanol. Haematoxylin and eosin (HE) staining and Masson trichrome staining were performed according to the manufacturer’s guidelines. Antigen repair was performed using a proteinase K solution (20 μg/mL) at 37 °C for 15 min. After blocking with Boxall, the sections were blocked with goat serum for 30 min and then incubated with anti-SMA (1:100; D4K9N; Stone CST) overnight at 4 °C. After washing with PBST, the sections were incubated with the HRP-bound secondary antibody (1:2000; ab97051; Abcam) for 1 h at room temperature. The cells were further counterstained with 3,3′-diaminobenzidine (DAB) and haematoxylin and visualized by microscopy.

### Tissue immunofluorescence analysis

Formalin-fixed and paraffin-embedded tissue sections were dewaxed with xylene and rehydrated by a gradient ethanol series. Antigen repair was performed in a 95 °C pressure cooker for 30 min using citrate buffer. Each section was blocked with 10% goat serum (16,210,064; Gibco) at 37 °C for 30 min and then incubated with the rat K10 primary antibody (1:200; 3C2F5; Novus). After overnight incubation at 4 °C, the sections were washed with PBST and incubated for 1 h with a secondary antibody labelled with Alexa Fluor 594 (1:200; ab150116; rat IgG against Abcam). DAPI was added dropwise to the sections, and the sections were then incubated while protected from light for 5 min and then subjected to four 5-min rinses with PBS. The remaining liquid in the dish was aspirated, the plates were sealed with an anti-fluorescence quencher, and the sections were taken using Zeiss LSM880, and the setting channel was FITC and DAPI. The acquisition software was Zeiss ZEN (black edition).

### Statistical analysis

Statistical comparisons between conditions were performed by one-way ANOVA or Student’s t test when appropriate. The results are presented as the means ± standard deviations, and differences were considered significant if *P* < 0.05: *P < 0.05, **P < 0.01, and ***P < 0.001.

## Results

### Morphology and characterization of the non-cross-linked ADM

**Fig. 1:**
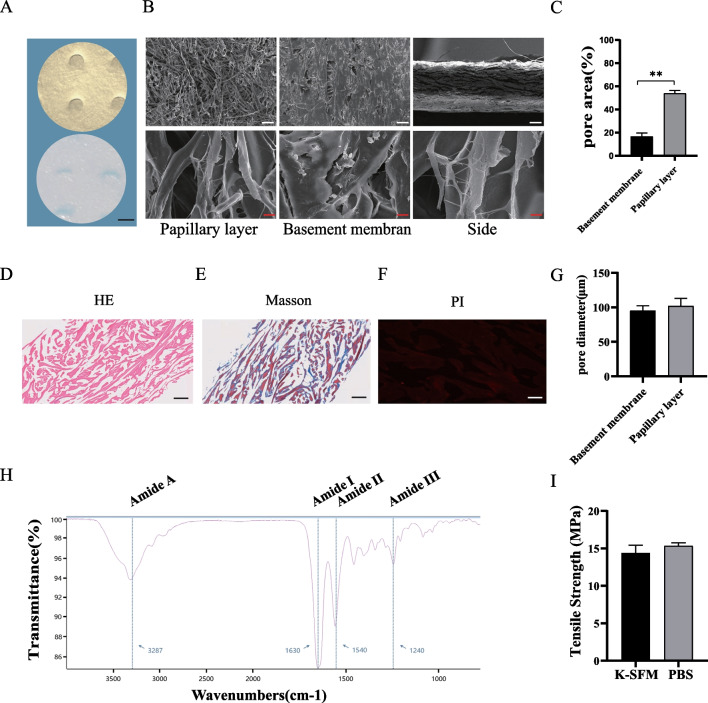
Physicochemical properties of the non-cross-linked ADM. **A** Gross view of freeze-dried ADM and ADM soaked in PBS; scale bar: 5 mm. **B** Scanning electron micrographs of ADM. White scale bar:100 μm; red scale bar: 2 μm. **C** Histogram of the porosity of the basement membrane and papillary dermis of the ADM (*n* = 3). **D–F** HE, Masson, and propidium iodide staining of non-cross-linked ADM; scale bar: 100 μm. **G** Histogram of the pore diameter of the basement membrane and papillary dermis of the ADM. **H** Components of the ADM patch were identified by FTIR. Collagen structures can be analysed by amide A (3287 cm^−1^), which reflects the stable triple helix structure formed by hydrogen bonds and amide I (1630 cm^−1^), amide II (1540 cm^−1^), and amide III (1240 cm^−1^), which reflect the secondary structure of collagen (triple helix structure). **I** Tensile strength control of ADM in PBS and K-SFM (n = 3). *P < 0.05, **P < 0.01, ***P < 0.001

Decellularized freeze-dried non-cross-linked ADM is a milky white sponge that becomes soft after wetting (Fig. 1A). SEM scans of the basement membrane, and papillary layer of the ADM showed that the natural ADM had a multilayer pore structure and an irregular pore distribution with different pore sizes (Fig. 1B, G). The porosity of the papillary layer was significantly higher than that of the basement membrane (*P* < 0.01), and trace amounts of mesh fibres and elastic fibres were detected (Fig. 1B). HE, Masson, and PI staining revealed no cellular components, cell debris, or nuclei (Fig. 1D, E and F). FTIR revealed the main absorption bands of peptide bonds in collagen (Fig. 1H), which included the N–H stretching vibration of amide A at 3287 cm^−1^, the C=O stretching of amide I at 1630 cm^−1^, the N–H bending of amide II at 1540 cm^−1^, and the C–N stretching of amide III at 1240 cm^−1^, which mainly derived from the H–N stretch vibration. Amide I, amide II, and amide III reflect the secondary structure (triple helix structure) of collagen, whereas amide A reflects hydrogen bond formation for stabilizing the triple-helical structure [24, 25]. The mean tensile strength of the ADM samples obtained in PBS and K-SFM was 15.20 and 14.63 MPa, respectively (Fig. 1I).

### Identification of EpSCs and engraftment of EpSCs in ADM

**Fig. 2:**
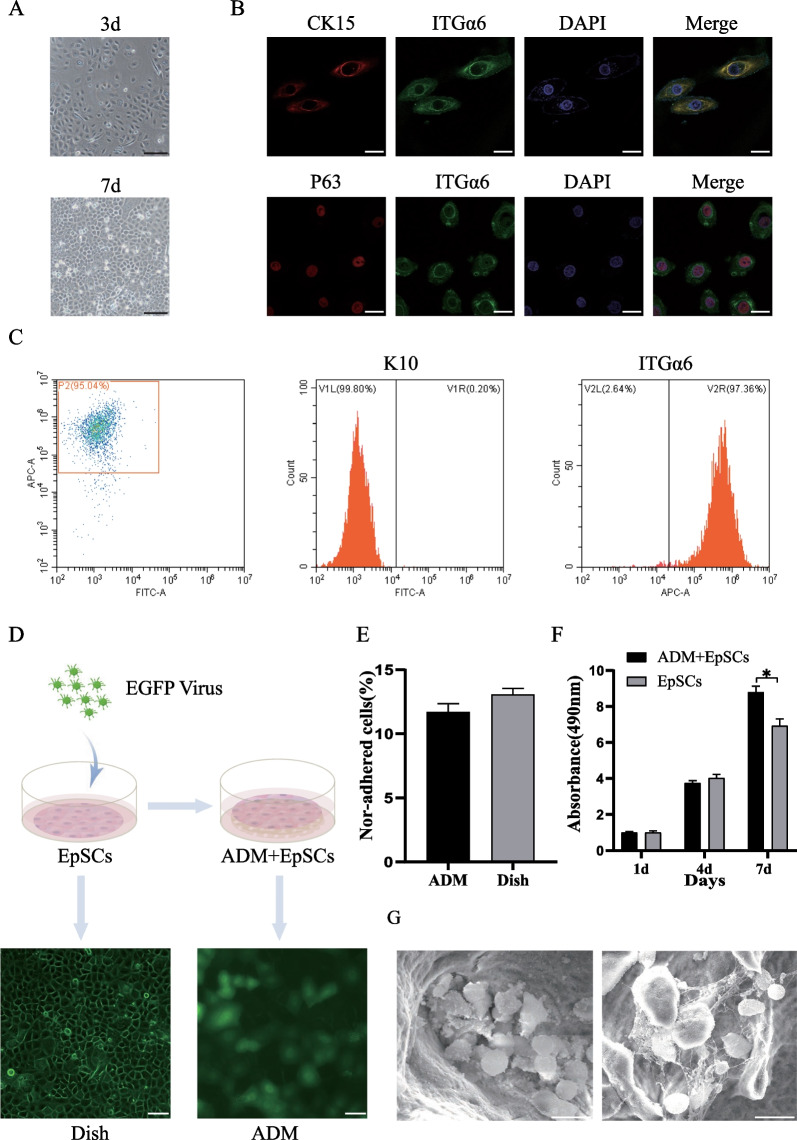
Identification, lentivirus transfection, and activity of EpSCs. **A** Rat EpSC morphology on the 3rd and 7th days; scale bar: 100 μm. **B** Identification of third-generation cells using p63, ITGα6, and CK15; scale bar: 100 μm. **C** Flow cytometry detection of the proportion of K10- and ITGα6-positive cells. **D** EpSCs were transfected with lentivirus to stably express EGFP on Petri dishes and in ADM sheets, scale bar: 50 μm. **E** Nonadherent cells after 24 h of the plating of EpSCs on Petri dishes and in ADM sheets. **F** Cell activity assay (MTT) of EpSCs on Petri dishes and in ADM sheets. **G** Scanning electron micrographs of EpSCs cultured in ADM. Scale bar: 20 μm

In our study, the isolated primary rat EpSCs had a circular shape, a small size and a robust refractive index. After 3 days of incubation in vitro, EpSCs formed clonal colonies and firmly adhered. After 7 days of culture, the cells proliferated significantly and became connected in paver-like sheets (Fig. 2A). EpSCs were passaged and amplified, and third-generation cells were analysed by immunofluorescence. Cells expressing p63, ITGα6, and CK15 were considered positive (Fig. 2B). Flow cytometry analysis showed that 95.04% of the cells were negative for K10 and positive for ITGα6 (Fig. 2C).

For lineage tracing, third-generation EpSCs were transfected with EGFP lentivirus and stably expressed green fluorescence. After EpSCs were seeded on the ADM sheet, confocal microscopy showed that EpSCs were evenly distributed in the ADM structure and migrated to the deep layer (Fig. 2D). We showed that at 24 h, the ADM matrix supported EpSC adhesion and proliferation and retained approximately 87% of the seeded cells (Fig. 2E). When measuring the activity of cells that adhered to the ADM using MTT, both absorbance activities increased with increases in the incubation times. After 7 days, the EpSCs cultured with ADM scaffolds, exhibited significantly more activity than those cultured in flat dishes (Fig. 2F). Moreover, we determined whether ADM was an adequate scaffold for EpSC transplantation by seeding EpSCs on the biomaterial and observing them by SEM. After 7 days of the coculture of EpSCs with ADM, SEM showed a large number of cells inside the ADM, and EpSCs attached to the matrix via various focal adhesion contacts (Fig. 2G).

### Effect of EpSCs on rat vascular endothelial cells

**Fig. 3:**
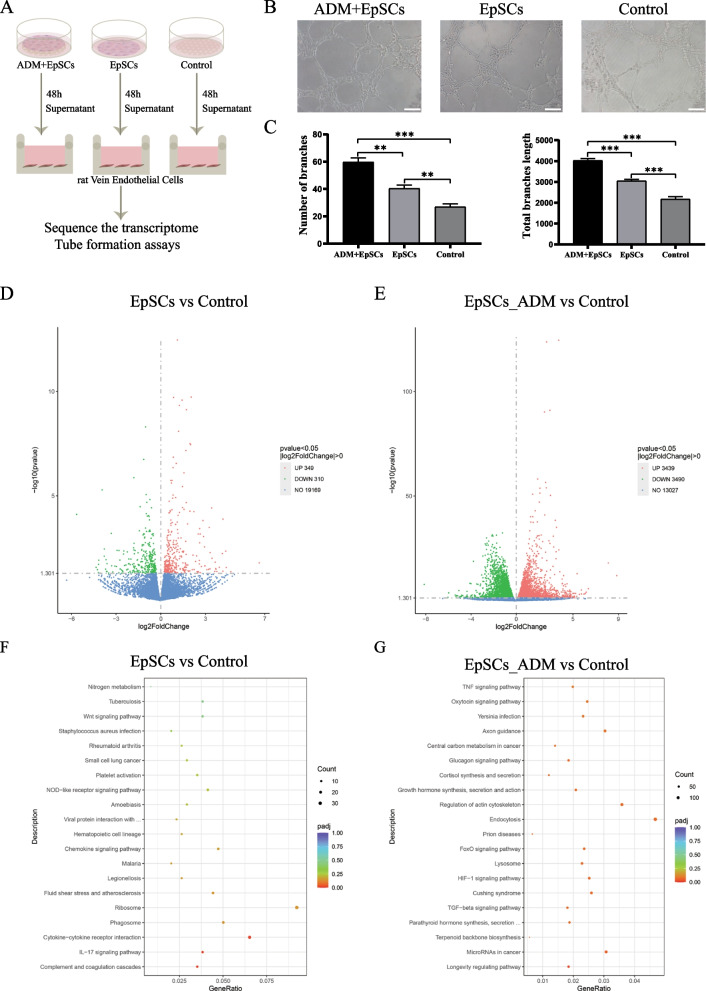
Proangiogenic effects and RNA-seq of KEGG pathways enriched in RVECs exposed to the supernatant of EpSCs. **A** Patterns of EpSCs under different culture conditions. The ADM + EpSCs group represents the EpSCs cultured in the ADM sheets, and the EpSCs group represents the EpSCs cultured on the dish. KFSM and ADM were used as the control groups. **B** Representative images of the tube formation ability of rat VECs stimulated with the supernatant under different conditions. Scale bar: 100 μm. **C** Quantification of tubule formation results. **D** Volcano plot of EpSCs vs. the control group. **E** Volcano plot of the EpSC + ADM group vs. the control group. **F** Kyoto Encyclopedia of Genes and Genomes (KEGG) pathway analysis of EpSCs vs. the control group. **G** Kyoto Encyclopedia of Genes and Genomes (KEGG) pathway analysis of the EpSC + ADM group vs. the control group

To assess the ability of the supernatant from EpSCs cultured in ADM scaffolds and on Petri dishes to promote angiogenesis in vitro, a tube formation assay was performed (Fig. 3A). The ADM + EpSC group showed a greater number of branches per field and a longer length of tubules in networks of RVECs compared with the control and EpSC groups. Interestingly, supernatant from the EpSC group also exhibited increased sprouting compared with that from the control group (Fig. 3B and C). Overall, the supernatant from EpSCs cultured with ADM had a more positive effect on angiogenesis than that from EpSCs alone.

To explore the mechanism through which EpSCs promote angiogenesis, we performed transcriptome sequencing to analyse the differentially expressed genes associated with endothelial cell function. Differential gene expression analysis revealed 349 significantly upregulated genes and 310 significantly downregulated genes in the EpSC group compared with the control group. However, the EpSC + ADM group had 3439 significantly upregulated genes and 3490 significantly downregulated genes compared with the control group (3D, E). To further explore the pathways involving these differentially expressed genes, a Kyoto Encyclopedia of Genes and Genome (KEGG) pathway analysis was conducted. The DEGs in the EpSC group compared with the control group were mainly enriched in “cytokine‒cytokine receptor interaction”, “IL-17 signalling pathway”, and “complement and coagulation cascades” (Fig. 3F). However, the DEGs in the EpSC + ADM group compared with the control group were mainly enriched in “TNF signalling pathway”, “regulation of actin cytoskeleton”, “endocytosis”, “FoxO signalling pathway”, “HIF-1 signalling pathway”, “TGF-beta signalling pathway”, and “longevity regulating pathway” (Fig. 3G).

Among the above-mentioned KEGG pathways, the “IL-17 signalling pathway”, “FoxO signalling pathway”, “TGF-beta signalling pathway”, and “HIF-1 signalling pathway” are reportedly involved in angiogenesis. These results indicate that the EpSC + ADM group showed more transcriptome-level changes in vascular endothelial cells than the EpSC group and that the differentially expressed genes were enriched in more pathways related to angiogenesis, which partly explains the superior proangiogenic function of the EpSC + ADM group.

### EpSCs improved wound healing and the survival of one-step tissue-engineered skin grafts

**Fig. 4:**
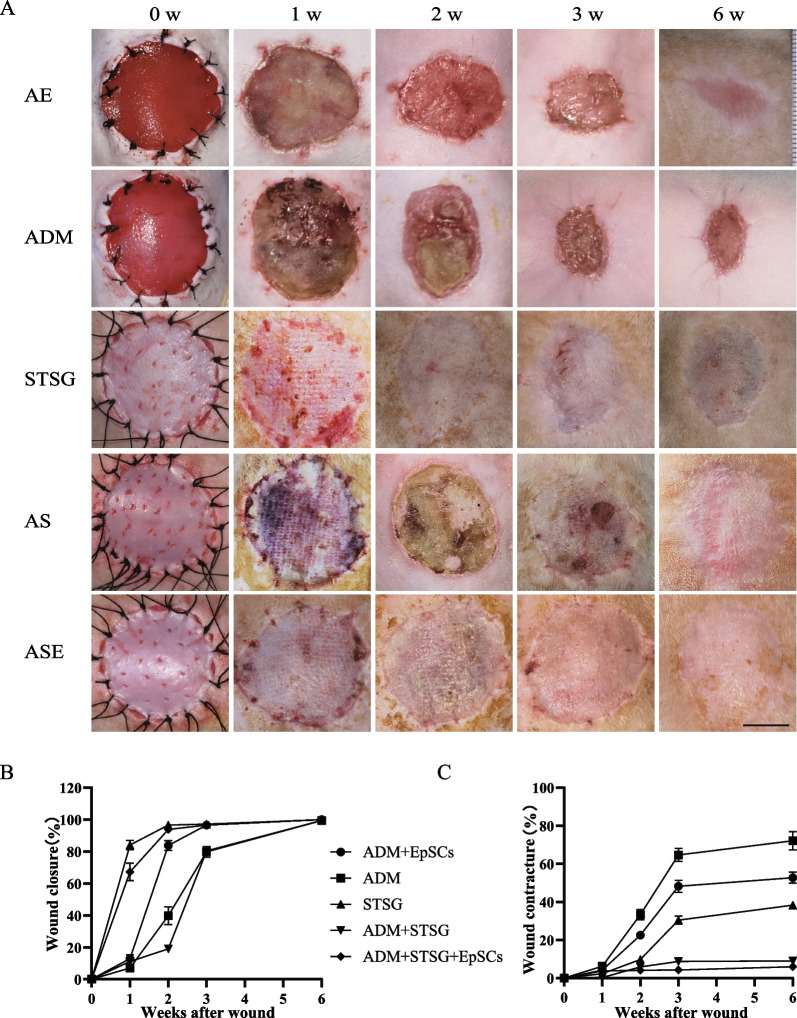
Healing and contraction of different skin grafts. **A** Morphological changes occurring in the diverse types of skin grafts (i.e. ADM, ADM + EpSCs (AE), STSG, ADM + STSG (AS), ADM + STSG + EpSCs (ASE)) over six weeks postsurgery, scale bar: 10 mm. **B** Wound closure rates of different skin graft groups at postinjury weeks 0, 1, 2, 3, and 6 postinjury. **C** Rates of wound contracture in the different skin graft groups at weeks 0, 1, 2, 3, and 6 postinjury. *P < 0.05, **P < 0.01 and ***P < 0.001

To observe the effect of EpSCs in vivo, the AE group showed a faster epithelial healing rate and higher healing quality than the ADM group, as indicated by epidermal crawling and epithelial healing (Fig. 4A, B). However, significant wound contractures of up to 50% were observed in both groups at 6 weeks after grafting (Fig. 4A, C). The one-step skin graft wound closure in the ASE group was significantly better than that in the AS group. The wound was almost unclosed, and some skin in the AS group was necrotic, with local ulceration and signs of instability and immaturity until 3 weeks (Fig. 4A, B). The skin graft remaining in the STSG group at 6 weeks indicated severe skin shrinkage. Nevertheless, no significant contractures were observed in either the AS or ASE group (Fig. 4C).

### EpSCs improved wound proliferation, epithelialization, and collagen remodelling

**Fig. 5:**
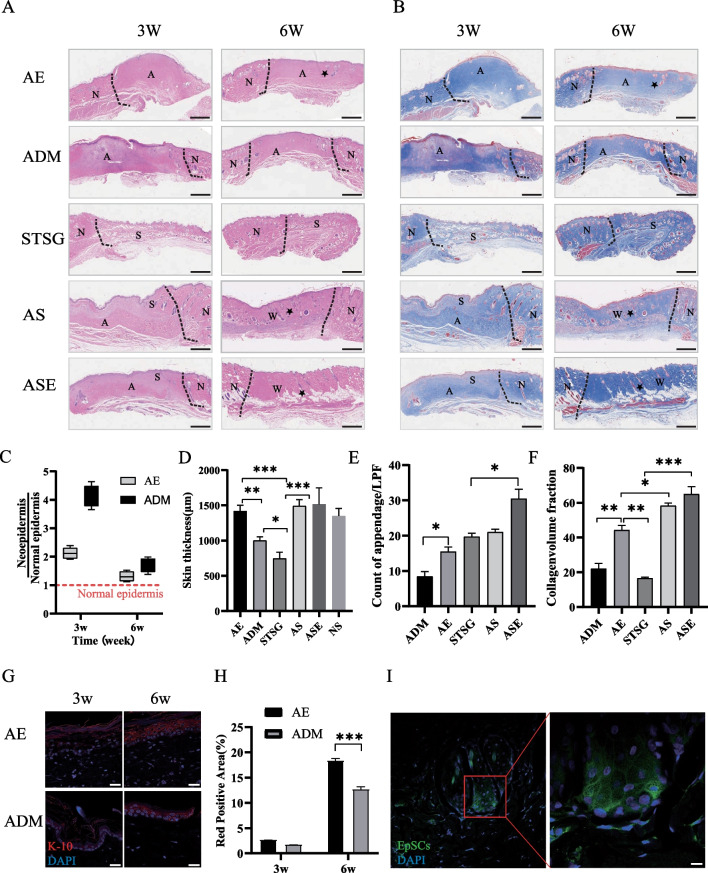
EpSCs improved ADM wound healing and promoted the survival of one-step tissue-engineered skin grafts. **A**, **B** HE and Masson staining of the skin. Scale bar: 1 mm. The black dotted line delimits its borders. A indicates acellular dermal matrix (ADM), N indicates normal skin, S indicates STSG skin, and W indicates wound skin.* indicates a hair follicle. **C** Ratio of the new epidermis thickness to the normal epidermis thickness. The red dotted line shows the thickness of the normal epidermis. **D** Skin thickness of each group at 3 weeks after surgery (*n* = 3). **E** Count of skin appendages/LPF. **F** Collagen volume fraction (CVF) of each group at 3 weeks after surgery (*n* = 3). **G** Comparison of K10 staining in AE and ADM groups 3 and 6 weeks after surgery. K10:red, DAPI: blue. Scale bar: 50 μm. **H** K10-positive area (%) of ADM and AE groups. **I** Representation of rat EGFP-EpSCs in the hair follicle structures. *EpSCs* Green, *DAPI* Blue. Scale bar: 10 μm

The morphology and thickness of the epidermis and collagen changes within the graft were simultaneously assessed by HE and Masson staining. The AE group more rapidly approached the normal epidermis and granulation tissue thickness (Fig. 5A, C, D) and showed more uniform collagen remodelling (Fig. 5B, F) and more skin attachment than the ADM group (Fig. 5A, E). We also found that EpSCs expressing EGFP could be colonized in the rat wound as well as the ADM (Additional file 1: Fig. S2A, B) and differentiate into hair follicles (Fig. 5I), and promote wound epithelialization (Fig. 5G, H). The skin slices of the STSG group were significantly atrophic and thinner and presented many necrotic foci and missing sebaceous glands. The grafts of the ASE group retained most of the skin appendages and exhibited loose connective tissue. Moreover, the collagen remodelling volume in the ASE group was nearly average, indicating good healing of the skin sheets (Fig. 5A, B).

### EpSCs enhanced angiogenesis of dermal substitutes and vascularization of tissue-engineered skin grafts

**Fig. 6:**
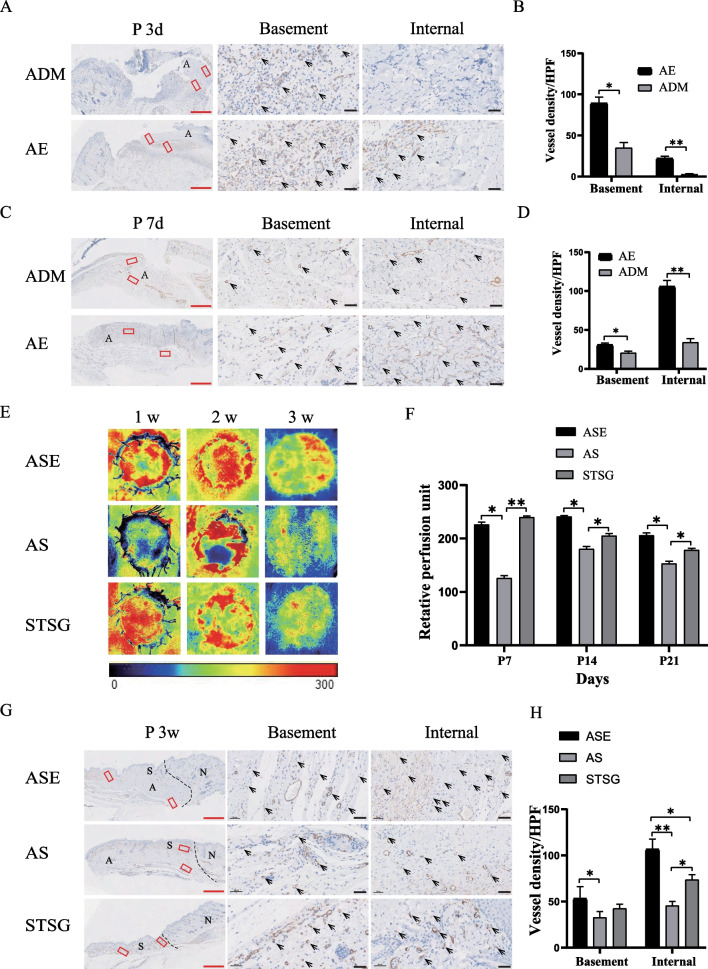
EpSCs enhanced the vascularization of dermal substitutes and novel tissue-engineered skin. **A**, **B** Representative area and analysis of wound tissue sections subjected to α-SMA staining on postinjury day 3 revealed microvascular regeneration in rat wounds in the ADM group and AE group. **C**, **D** Representative area and analysis of wound tissue sections subjected to α-SMA staining on postinjury day 7 in the ADM group and AE group. **E** Representative blood perfusion images of different skin graft groups at 1, 2, and 3 weeks after surgery. **F** Relative perfusion unit values at 1, 2, and 3 weeks (*n* = 3). **G**, **H** Representative area and analysis of wound tissue sections subjected to α-SMA staining at week 3 postinjury show microvascular regeneration in rat wounds of the ASE, AS, and STSG groups. A indicates acellular dermal matrix (ADM), N indicates normal skin, and S indicates STSG skin. The arrows indicate the microvasculature. Red scale bar: 1 mm, white scale bar: 50 μm

For the assessment of angiogenesis within the ADM, immunohistochemical staining for α-SMA in mature vessels was performed. At 3 and 7 days, the AE group had more vessels in both the base and interior of the ADM than the ADM group. Mature angiogenesis was present within the almost full-thickness ADM at 7 days (Fig. 6A, B, C, D). These results suggested that EpSCs could promote rapid angiogenesis and vascular maturation in ADM in vivo.

Blood perfusion on the skin surface was observed at weekly intervals after skin grafting, and a weak blood flow signal was observed in the ASE and autologous STSG groups at 1 week. No significant differences in the blood perfusion signal were found between the ASE and autologous STSG groups at 2 weeks and 3 weeks postsurgery in rats. Nevertheless, the two groups were significantly different from the AS group. The above-mentioned results macroscopically showed that the blood supply of skin slices in the ASE group within 3 weeks after surgery was better than that found for the AS group, which indicated that EpSCs promoted the survival of STSG skin slices on the surface of the ADM (Fig. 6E, F). These findings suggest that the visible colour may be related to angiogenesis. For the assessment of angiogenesis, immunohistochemical staining for α-SMA was performed 3 weeks after surgery, and the number of vessels in both the ADM base and interior in the ASE group was significantly higher than that in the AS group (Fig. 6G, H).

## Discussion

EpSCs maintain skin homeostasis and participate in repair following injury. Recently, the use of EpSCs for wound healing has attracted the attention of researchers. In addition, advances in tissue engineering have increased the interest in applying EpSCs in tissue-engineered scaffolds to reconstitute injured tissues. The combination of biomaterial scaffolds with stem cells is a potential strategy for engineering tissues, delivering cells, and regenerating native organs [26]. In this study, EpSCs were extracted, and native non-cross-linked ADM sheets were prepared. These materials had structurally porous structures while retaining the basement membrane surface; thus, these materials retained cell adhesion sites and did not release cytotoxic degradation products, which facilitates the adhesion and homing of epidermal stem cells for subsequent expression and repair. This study used ADM sheets as the culture microenvironment for EpSCs and thus creates a dynamic microenvironment of cell-to-matrix interactions.

We subsequently validated the effects on wound repair in vivo. Many stem cell-related studies have been performed in rodents [27] , where multiple skin graft models have been used. In this study, a large wound (approximately 5 square centimetres in area) was designed to observe the effect of tissue engineering skin grafts on wound repair to mimic the healing process of wound repair in humans, and a rat model with immune characteristics and a larger volume was selected. However, full-thickness skin defect wounds in rats without skin grafting treatment healed through contracture of the subcutaneous flesh membrane, which is inconsistent with the wound healing process in humans, so STSGs were used as a control group for wound skin grafting repair, and the negative control group evaluated the natural rate of wound healing and was not considered in this study. Full-thickness excisional wounds with a diameter of 2.5 cm were created in the study. For similar large wounds, EpSCs can promote ADM wound epithelial healing and the growth of skin appendages and hair follicles. Nevertheless, the wound healing time was unfortunately longer than 3 weeks, and contracture was severe. The toughness of the appearance of epithelialized skin differed significantly from that of the surrounding normal skin, which is far from the standards for clinical application. Many studies using stem cells for wound therapy have achieved promising results [5, 6, 28–31]. As skin tissue-specific stem cells, EpSCs are closely related to skin regeneration. Our previous study found that EpSCs could promote wound healing and increase the number of wound blood vessels [23]. However, further mechanistic studies have not yet been conducted. Here, we observed increased biological activity, particularly concerning vascularization effects, in the EpSC + ADM group. To further explore the mechanism, the active substances produced during angiogenesis were secreted by EpSCs in the ADM environment, as revealed by tube formation experiments and transcriptome sequencing analysis. The active factors secreted by EpSCs cultured in ADM structures can affect the “FoxO signalling pathway”, “TGF-beta signalling pathway”, and “HIF-1 signalling pathway” of vascular endothelial cells, which are some of the main pathways affecting angiogenesis [32, 33]. Therefore, we suggest that the complex interplay between EpSCs within the skin and the ADM niche provides important ideas for skin regeneration and reconstruction.

To date, no engineered skin tissue can permanently cover full or deep dermal wounds during one-stage surgery. Furthermore, most available skin alternatives include only fibroblasts and keratinocytes and are insufficient to recapitulate the complexity of natural skin. Plastic and repair surgery for ADM and STSGs are often selected in the clinic, particularly for wounds near functional sites such as joints. A dermal stent is used first to cover the injury, and after 2 to 4 weeks, the dermal stent allows the wound to establish sufficient blood transport before the second phase of STSG transplantation; this is a common approach for two-step skin grafting. Two-step skin grafting in a patient requires a long time in the hospital and has high costs. Moreover, the lack of natural skin accessory structures, such as hair follicles, sebaceous glands, and sensory nerve receptors, is also a problem for clinicians and patients [11, 34–36]. A lack of early and appropriate vascularization causes graft necrosis or loosening of implanted skin substitutes, which ultimately leads to poor skin implantation [37, 38]. We found that rat EpSCs promoted vascularization at the base and interior of the ADM in rats. By 1 week after surgery, mature vessels were entirely generated within a 4-mm thickness, which significantly shortened the time of 2-4 weeks for complete vascularization compared with conventional ADM implantation. The transplanted STSGs could supply nutrients and oxygen through the plasma within 1 week, which can maintain the survival of the skin graft [39].

The above-described findings prompted us to attempt to construct new tissue-engineered skin using a combination of ADM, EpSCs, and STSGs for a single-stage procedure to repair full-thickness wounds in rats. The survival of skin slices in the ASE group was significantly superior to that in the AS group and close to that in the STSG group. This finding may have been obtained because the initial superficial STSG survival largely depended on nutrient diffusion from the implanted early wound bed and timely reconstruction of functional blood vessels after 1 week to supply nutrients and oxygen. Moreover, non-invasive detection of blood perfusion in skin grafts was performed at the early stage. The results indicated that a blood flow signal had appeared in all the skin graft areas of rats successfully transplanted 1 week after surgery, and the blood flow of the rats in the ASE group was better than that in the AS group at 2 and 3 weeks. Through vascular staining, we found that the number and area of microvessels was significantly increased in the ASE group at the early stage. Therefore, a more significant number of vessels is critical for enhancing the maintenance and survival of the graft, particularly at the initial phase.

Regarding skin patch shrinkage, the STSG group began to show skin graft shrinkage approximately 7 days after surgery, and excessive fibrosis, characterized by skin shrinkage and abnormal morphology, was observed. The ASE group had the lowest shrinkage rate at 42 days, with only a 13% reduction, and appeared close to the surrounding normal skin. The contraction rate of AS skin pieces was significantly greater than that of the ASE group starting from 14 days after surgery. This effect may cause necrotic contracture due to suboptimal healing of skin pieces. EpSCs are simultaneously able to improve the quality and survival of skin sheets, possibly due to a series of wound healing events, including the inflammatory response, neovascularization, proliferation, collagen deposition, and remodelling [40]. Our results suggest that timely vascularization of the embedded ADM sheets can reduce the contracture of superficial STSGs.

Hair follicles, sebaceous glands, and dermal collagen are essential components for the reconstruction of skin structure and function. Seven days after implantation, the number of skin appendages, particularly sebaceous glands, in the AS grafts was significantly reduced. Moreover, degradation of implanted collagen fibres or necrosis occurred, and these phenomena may have been caused by an insufficient blood supply. The performance was significantly improved in the ASE group compared with the other groups. This result may be related to the ability of vascularization promoted by EpSCs to improve the blood supply and the fact that EpSCs can differentiate to form skin attachments such as hair follicles.

This study has some limitations. Although the new tissue-engineered skin achieved one-step transplantation and the skin graft survived satisfactorily after transplantation, the obtained EpSCs require a long time of culture *in vitro*, and this step is followed by allogeneic EpSC transplantation. In addition, the proangiogenic effects may be mainly derived from the paracrine activity of EpSCs, which dominated the healing process in our experiments. Further studies are needed to confirm this hypothesis. The observation period in the study was only 6 weeks, and longer implantation time points are needed to evaluate the complete remodelling process of dermal substitutes *in vivo*.

## Conclusion

Our study shows that rat EpSCs can promote neovascularization in dermal replacement because rat EpSCs can promote neoangiogenesis through paracrine activities such as secreted vasoactive factors. In this study, a new type of tissue-engineered skin was further constructed. Autologous STSGs combined with EpSCs and ADM realized one-step repair of a full-thickness wound with minimal skin shrinkage, skin accessories were generated, and the morphology was close to that of normal skin. The use of a combination of EpSCs, ADM scaffolds, and STSGs provides a promising regenerative strategy for skin engineering, stem cell delivery, and regeneration of damaged skin tissues. This conclusion offers a theoretical basis for the mechanism through which EpSCs promote the survival of transplanted dermal substitute skin grafts and provides a new idea for clinical one-step compound skin grafting.

## Supplementary Information

**Additional file 1: Fig. S1. A** A 25-mm-diameter round of STSGs was obtained from the abdominal skin of autologous rats. The diameter is equal to the size of the dorsal wound. **B** The thickness of STSGs removed from the abdomen of rats was 0.27 mm as measured by a Vernier calliper. **C** The thickness of ADM sheets was 0.36mm as measured by a Vernier calliper. **D** A 25-mm-diameter round full-thickness skin defect was made on the dorsal side of the rat. **E** A 25-mm-diameter round ADM sheet was implanted to cover the wound defects. **F**, **G** Epidermal stem cells were sprayed on both sides of the ADM. **H**, **I** The ADM and STSG were fixed with intermittent sutures of 5-0 nylon silk thread in the wound. **J** The transplanted ADM and STSG were fixed by packaging and compression. **Fig. S2.** Representation of different magnifications of rat EGFP-EpSCs in the rat wound as well as the ADM at 3 weeks after surgery in vivo. **A** ADM indicates acellular dermal matrix, and the white line delimits its borders. EpSCs-EGFP: green; DAPI: blue. Scale bar: 200 μm. **B** The red arrows indicate the EpSCs-EGFP in ADM. Scale bar: 50 μm.

## Data Availability

The sequencing data have been deposited in the National Center for Biotechnology Information (NCBI) Sequence Read Archive (SRA) database under the study accession number PRJNA880693 and PRJNA880715. The data supporting this study’s findings are available from the corresponding author upon reasonable request.

## Abbreviations

EpSCs: Epidermal stem cells
ADM: Acellular dermal matrix
STSGs: Split-thickness skin grafts
FTSGs: Full-thickness skin grafts
ECM: Extracellular matrix
EGFP: Enhanced green fluorescent protein
MTT: 3-(4,5-Dimethyl methyl thiazole-2-yl)-2–5-diphenylbromotetrazole method
RNA-seq: RNA sequencing analysis
RVEC: Rat vein endothelial cell
KEGG: Kyoto Encyclopedia of Genes and Genomes
RPU: Relative perfusion unit
K-SFM: Keratinocyte serum-free medium
FN: Fibronectin
VEGF: Vascular endothelial growth factor
