# Supplementary figures and images for: Single-stage transplantation combined with epidermal stem cells promotes the survival of tissue-engineered skin by inducing early angiogenesis
Source: Stem Cell Res Ther. 2023 Mar 23;14:51. doi: 10.1186/s13287-023-03281-z (PMC10035248; doi:10.1186/s13287-023-03281-z)

Figure S1

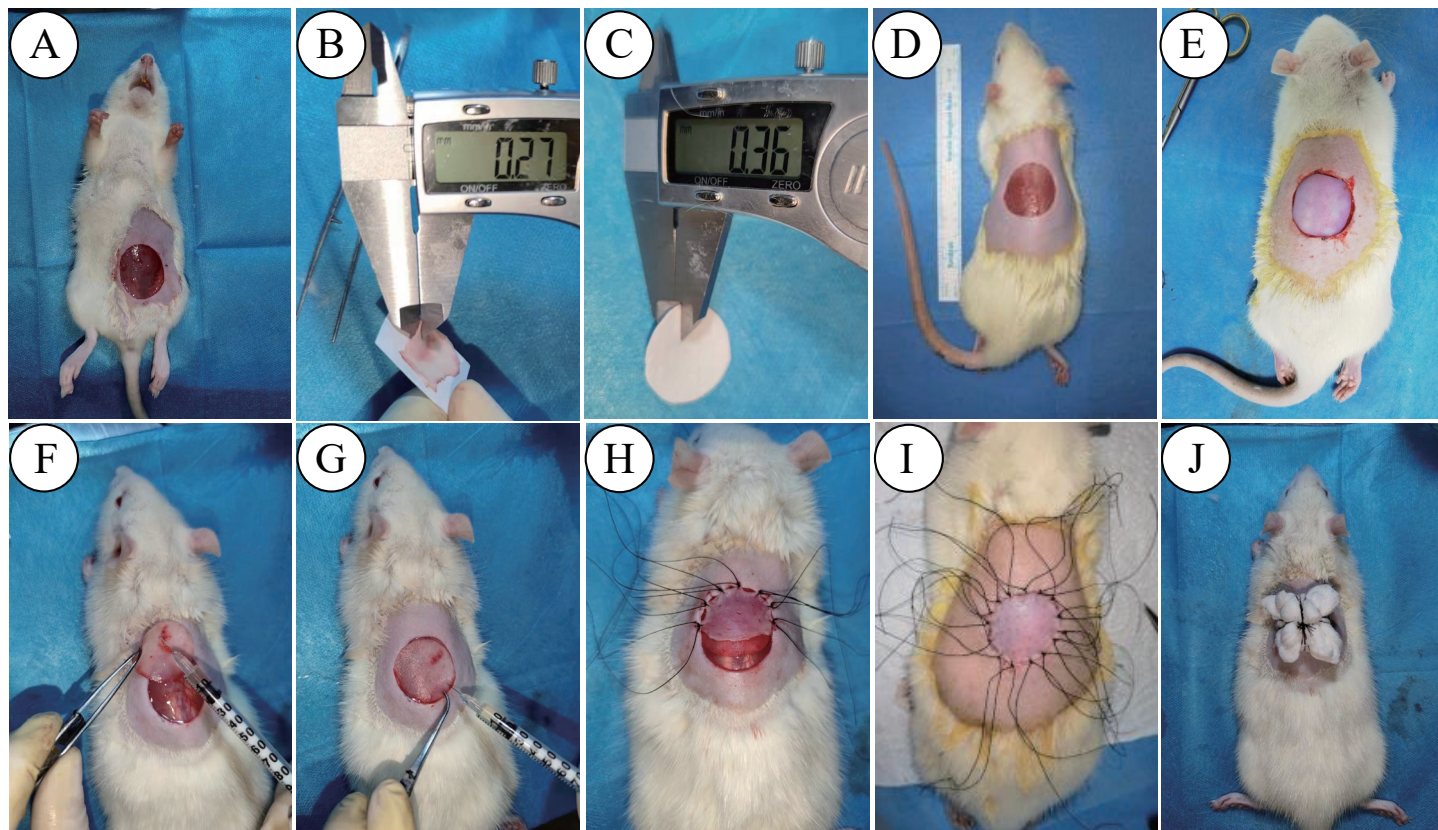

Figure S2

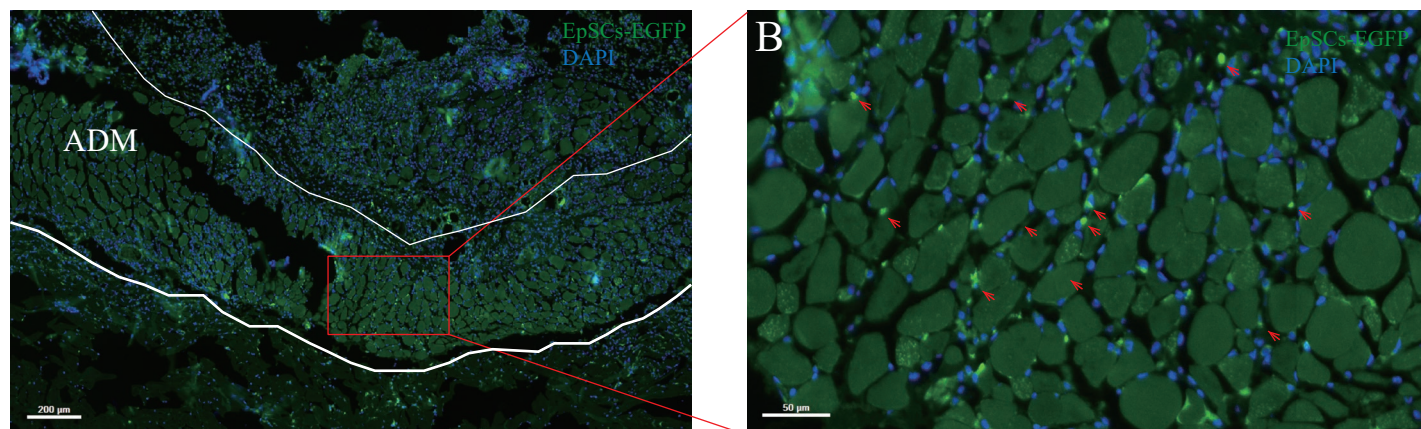

Supplement: Supplementary file 1 — Additional file 1: Fig. S1. A A 25-mm-diameter round of STSGs was obtained from the abdominal skin of autologous rats. The diameter is equal to the size of the dorsal wound. B The thickness of STSGs removed from the abdomen of rats was 0.27 mm as measured by a Vernier calliper. C The thickness of ADM sheets was 0.36mm as measured by a Vernier calliper. D A 25-mm-diameter round full-thickness skin defect was made on the dorsal side of the rat. E A 25-mm-diameter round ADM sheet was implanted to cover the wound defects. F, G Epidermal stem cells were sprayed on both sides of the ADM. H, I The ADM and STSG were fixed with intermittent sutures of 5-0 nylon silk thread in the wound. J The transplanted ADM and STSG were fixed by packaging and compression. Fig. S2. Representation of different magnifications of rat EGFP-EpSCs in the rat wound as well as the ADM at 3 weeks after surgery in vivo. A ADM indicates acellular dermal matrix, and the white line delimits its borders. EpSCs-EGFP: green; DAPI: blue. Scale bar: 200 μm. B The red arrows indicate the EpSCs-EGFP in ADM. Scale bar: 50 μm. [file 13287_2023_3281_MOESM1_ESM.pdf]
